# Radiomics Analysis Based on Diffusion Kurtosis Imaging and T2 Weighted Imaging for Differentiation of Pancreatic Neuroendocrine Tumors From Solid Pseudopapillary Tumors

**DOI:** 10.3389/fonc.2020.01624

**Published:** 2020-08-21

**Authors:** Yan-Jie Shi, Hai-Tao Zhu, Yu-Liang Liu, Yi-Yuan Wei, Xiu-Bo Qin, Xiao-Yan Zhang, Xiao-Ting Li, Ying-Shi Sun

**Affiliations:** Department of Radiology, Key Laboratory of Carcinogenesis and Translational Research, Peking University Cancer Hospital, Beijing, China

**Keywords:** pancreatic neoplasms, neuroendocrine tumor, radiomics, magnetic resonance imaging, tumor imaging and diagnosis

## Abstract

**Objective:**

To develop and validate a radiomics model of diffusion kurtosis imaging (DKI) and T2 weighted imaging for discriminating pancreatic neuroendocrine tumors (PNETs) from solid pseudopapillary tumors (SPTs).

**Materials and Methods:**

Sixty-six patients with histopathological confirmed PNETs (*n* = 31) and SPTs (*n* = 35) were enrolled in this study. ROIs of tumors were manually drawn on each slice at T2WI and DWI (*b* = 1,500 s/mm^2^) from 3T MRI. Intraclass correlation coefficients were used to evaluate the interobserver agreement. Mean diffusivity (MD) and mean kurtosis (MK) were derived from DKI. The least absolute shrinkage and selection operator regression were used for feature selection.

**Results:**

MD and MK had a moderate diagnostic performancewith the area under curve (AUC) of 0.71 and 0.65, respectively. A radiomics model, which incorporated sex and age of patients and radiomics signature of the tumor, showed excellent discrimination performance with AUC of 0.97 and 0.86 in the primary and validation cohort. Moreover, the new model had better diagnostic performance than that of MD (*P* = 0.023) and MK (*P* = 0.004), and showed excellent differentiation with a sensitivity of 95.00% and specificity of 91.67% in primary cohort, and the sensitivity of 90.91% and specificity of 81.82% in the validation cohort. The accuracy of radiomics analysis, radiologist 1, and radiologist 2 for diagnosing SPTs and PNETs were 92.42, 77.27, and 78.79%, respectively. The accuracy of radiomics analysis was significantly higher than that of subjective diagnosis (*P* < 0.05).

**Conclusions:**

Radiomics model could improve the diagnostic accuracy of SPTs and PNETs and contribute to determining an appropriate treatment strategy for pancreatic tumors.

## Introduction

Pancreatic neuroendocrine tumors (PNETs) and solid pseudopapillary tumors (SPTs) are increasingly encountered in the course of routine radiology practice due to greater diagnostic capability of imaging techniques. Because the clinical management and patient prognosis significantly differ between these two major pancreatic lesions, accurate and timely imaging diagnosis is essential (1, 2). SPTs have a low malignant potential with an excellent prognosis following complete resection; metastases are uncommon for SPTs (2–4). PNETs have malignant behavior and worse prognosis compared to SPTs. In addition, for PNETs, metastases, and vascular abutment or invasion are very common (1). Surgical resection, chemotherapy, or target treatment can be applied for patients with PNETs (2). Several criteria can aid in differentiating PNETs from SPTs. PNETs have low signal intensity at T1-weighted imaging, whereas SPTs, which contain hemorrhage, may have high signal intensity at T1-weighted imaging (5). Compared with SPTs, which show progressive enhancement, PNETs are more vascular and demonstrate diffuse or ringlike hyperenhancement on the arterial phase (5). However, PNETs may demonstrate hypo-enhancement. Moreover, calcification and cystic degeneration are commonly present in both tumors. Therefore, differentiating PNETs from SPTs based on imaging manifestation may be challenging when the atypical characteristics are found.

Jang et al. found that higher mean value of tumor-to-parenchyma ratio on arterial and portal phases is a useful MR imaging feature for diagnosing PNETs from SPTs and adenocarcinoma with an accuracy of 91.4% (6). However, the measurement signal intensity of pancreatic tumors is not a routine clinical practice. Driven by the “big data” trend, radiomics develops rapidly. Radiomics analysis can extract a large number of quantitative features from medical imaging to determine relationships between such features and the underlying pathophysiology. Radiomics with non-invasive and low-cost properties have been applied in medical imaging for pre-diagnosis assistance (7). Li et al. reported that texture analysis could be used to sensitively distinguish between non-functional PNETs and SPTs on MRI (8), which further confirms the clinical value of radiomics in differentiating major pancreatic lesions.

Diffusion kurtosis imaging (DKI) can provide a more accurate model of diffusion and capture the non-Gaussian diffusion parameters for tissue heterogeneity. DKI has been successfully applied for assessment of pancreas and pancreatic disease (9, 10). We speculated that DKI might provide more optimal identification characteristics for differentiation between PNETs and SPTs.

DKI and radiomics analysis may demonstrate the differences in heterogeneity, tumor microenvironment, and blood supply characteristics between PNETs and SPTs. In the present study, we examined a new radiomics-based model that integrated DKI and T2WI radiomics signature for differential diagnosis of PNETs and SPTs.

## Materials and Methods

### Patients

All patients were diagnosed with PNET or SPT. The inclusion criteria were the following: (1) patients underwent preoperative MRI using 3.0 T MR scanner, including DWI, T2WI, and contrast enhanced MRI; (2) PNETs and SPTs were histologically confirmed by surgery; (3) patients who did not receive chemotherapy or radiotherapy before surgery. The exclusion criteria were: (1) lack of DWI data and (2) insufficient MRI quality to obtain a measurement. Finally, thirty-one patients with PNET and thirty-five patients with SPT were finally analyzed from Jan 2011 to Dec 2018. The 66 lesions were allocated to primary and validation cohorts in a 2:1 ratio; the 44 lesions were allocated to the primary cohort and the 22 lesions to the validation cohort.

### MR Examination

All MRI examinations were performed on a 3.0T MR scanner (Discovery 750; GE Healthcare) using an 8-channel phased-array body coil in the supine position. The MRI sequences included T2-weighted single shot fat spin echo (SSFSE), fat-suppressed (FS) T2-weighted fast spin echo (FSE), in- and out-of-phase sequences, fat-suppressed (FS) T1-weighted with 3D Lava-flex sequence. DWI was performed with spin-echo, single-shot echo-planar imaging (EPI) sequence axially acquired prior to contrast administration with gradient factor of *b* = 0, 20, 50, 100, 200, 600, 800, 1,000, 1,200, and 1,500 s/mm^2^. The Integrated Parallel Acquisition Techniques (IPAT) imaging option with a factor of 3 and distortion correction were applied. Number of gradient directions of 3, echo spacing of 0.54 ms, bandwidth of ±250 kHz, and flip angle of 90°were used to DKI sequence. In order to maintain a sufficient signal-to-noise ratio, a different number of excitation (NEX) was chosen; the NEX of *b* = 0–800, 1,000, 1,200, and 1,500 s/mm^2^ were 1, 2, 4, and 6, respectively. Contrast-enhanced MRI was performed during the arterial phase (25 s), portal venous phase (60 s), and delayed phase (120 s) following intravenous injection. Contrast-enhanced MRI was performed using a breath-hold fat-suppressed 3D T1-weighted lava flex sequence. Intravenous injection of gadolinium-diethylenetriamine pentaacetic acid (DTPA) (Magnevist; Bayer Schering, Berlin, Germany) at a dose of 0.1 mmol/kg body weight and flow rate 2 ml/s was applied, followed by a 15-ml saline flush. All MRI scans were retrieved from the picture archiving and communication system for further image feature extraction. Detailed MR imaging parameters are summarized in Table 1.

**TABLE 1:** MRI protocol parameters.

Sequences	Orientation	TR/TE (ms/ms)	Matrix	NEX	Thickness/gap (mm)
T2WI (SSFSE)	Coronal	2,000/100	384 × 244	1	7/1
T2WI (FSE)	Axial	8,000/109	288 × 256	4	5/1
FS-T2WI (FSE)	Axial	8,000/109	288 × 256	4	5/1
DWI (EPI)	Axial	6,000/93.3	128 × 128	1–6	5/1
T1WI (lava flex)	Axial	3.2/2	256 × 192	1	5/-2.5
Arterial phase (lava flex)	Axial	3.2/1.5	256 × 192	1	5/-2.5
Portal phase (lava flex)	Axial	3.2/1.5	256 × 192	1	5/-2.5
Delayed phase (lava flex)	Axial/coronal	3.2/1.5	256 × 192	1	5/-2.5

*T2WI, T2 weighted imaging; T1WI, T1 weighted imaging; FS, fat-suppressed; SSFSE, single shot fast spin echo; FSE, fast spin-echo; EPI, echo-planar imaging; TR, repetition time; TE, echo time; NEX, number of excitation.*

### Image Analysis and Radiomics Feature Extraction

Pre-operation MRI was analyzed by two radiologists (Dr. Shi, with 10 years of experience in pancreatic tumors; Dr. Liu, with 8 years of experience in pancreatic tumors). The regions of interests (ROIs) were manually drawn with software of ITK-SNAP^1^ using DWI and T2WI data. The tumor was contoured slice by slice to obtain the entire neoplastic ROIs, which were placed on the high signal intensity region on DWI (*b*-value of 1,500 s/mm^2^) and T2WI on each slice. In case no high signal was detected on DWI compared with the normal pancreas, the ROIs were placed on the tumor region, as determined by T1WI, FS-T2WI, and contrast imaging.

Totally 195 features were extracted by the open-source codes designed by Vallières et al. (11), including 65 from T2-weighted image, 65 from D_*app*_ image, and 65 from K_*app*_ image. The 65 features were categorized into three groups as follows: (1) voxel-intensity computational features, (2) texture features, (3) shape features. The logistic regression model was trained with LASSO (least absolute shrinkage and selection operator) regularization. A fourfold cross-validation was used to determine the hyperparameter λ at the minimum mean area under the curve (AUC) of receiver operating characteristics (ROC). A signature was generated for each subject by the linear combination of selected features weighted by their coefficients.

Pre-operation MRI for subjective diagnosing SPTs and PNETs was based on combining the age, gender and symptom of patients, laboratory examinations and MRI features of pancreatic tumors independently evaluated by two experienced abdominal radiologists (Dr. Wei with 7 years of experience in pancreatic tumors, and Dr. Qin with 5 years of experience in pancreatic tumors). The maximum diameters of the tumors were independently measured by two radiologists, and the mean value was calculated.

### Parameter Estimation

After the ROIs delineation, DKI diffusion parameters were obtained using the following equation: $S\,\left(b\right)/S\,\left(0\right)=\exp\left(-b\cdot {\text{D}}_{\text{app}}+1/6\cdot b^{2}\cdot {\text{D}}_{\text{app}}^{2}\cdot {\text{K}}_{\text{app}}\right)$, where *S* was the signal intensity at a function of *b*, *S*(0) was a signal intensity at *b* = 0 s/mm^2^, *b* was a factor dependent on the pulse duration and strength of the diffusion gradients. D_*app*_ was the apparent diffusion coefficient (in mm^2^/s), and K_*app*_ was the apparent diffusion kurtosis coefficient. K_*app*_ and K_*app*_ were obtained from equation (12). All the DWI data were considered for DKI analysis.

### Interobserver Agreement

Interobserver reproducibility of ROIs detection and feature extraction were determined using the T2WI and DWI data of all patients for ROI-based radiomics feature generation blindly by Dr. Shi and Dr. Liu. Intraclass correlation coefficients were used to evaluate the interobserver agreement in terms of feature extraction. A coefficient of 0.81–1.00 indicated an almost perfect agreement; 0.61–0.80 was a substantial agreement; 0.41–0.60 was a moderate agreement; 0.21–0.40 was a fair agreement, and 0–0.20 indicated a poor or no agreement (13).

### Statistical Analysis

Student’s *t*-test was used to determine whether radiomics features were significantly different between PNETs and SPTs. Pearson Chi-square test was used to analyze qualitative data between PNETs and SPTs. Likelihood-ratio was used if any cell had expected count less than 5. AUC of different methods was compared by the method proposed by DeLong et al. Maximum Youden index (sensitivity + specificity - 1) was used to determine the cutoff value to separate PNETs and SPTs. *P*-value <0.05 was considered to be statistically significant.

## Results

### Patient Characteristics

A total of 31 patients with PENTs (16 men and 15 women; age range, 23–71 years; mean age, 53.20 ± 12.78 years) and 35 patients with SPTs (9 men and 26 women; age range, 15–68 years; mean age, 31.24 ± 13.41 years) were enrolled in this study. There were significant differences in age (*P* = 0.00) and gender (*p* = 0.00) between PNETs and SPTs groups. The mean maximum tumor diameters of the PNETs and SPTs were 38.67 ± 24.31 mm (range, 8–145 mm) and 47.42 ± 28.65 mm (range, 10–125 mm), respectively. There was no significant difference in size between the two types of pancreatic neoplasms (*P* = 0.19).

### Interobserver Agreement

Two radiologists independently delineated the ROIs of tumors, achieving satisfactory agreement. The ICC (intraclass correlation coefficient) of each feature extracted from the two delineations was calculated. The ICC of 65 features from T2 weighted images was 0.67 ± 0.27. The ICC of 65 features from D_*app*_ images was 0.81 ± 0.19. The ICC of 65 features from K_*app*_ images was 0.83 ± 0.23.

### DKI Parameters for the Diagnosis of SPTs and PNETs

Mean D_*app*_ (MD) and mean K_*app*_ (MK) were used to differentiate SPTs from PNETs. MD of the SPTs and PNETs were (2.11 ± 0.67) × 10^–3^ mm^2^/s and (2.36 ± 0.48) × 10^–3^ mm^2^/s, respectively. MK of the SPTs and PNETs were 0.91 ± 0.18 and 0.84 ± 0.15, respectively. No statistically significant difference was found in MD (*P* = 0.13) and MK (*P* = 0.10) between the two types of pancreatic neoplasms. The areas under the curves (AUCs) of MD and MK for diagnosing PNETs and SPTs were 0.71 (95% confidence interval (CI), 0.584–0.839), and 0.65 (95% CI, 0.511–0.783), respectively.

### Pre-operation Subjective Diagnosis of Abdominal MRI

The distribution of SPTs and PNETs among the patient population based on subjective MRI diagnosis and histopathological analysis was presented in Table 2. The sensitivity, specificity, positive predictive value (PPV), negative predictive value (NPV) and diagnostic accuracy of subjective MRI diagnosis for SPTs from PNETs by two radiologists were 80.0% (28/35) and 82.86% (29/35), 74.19% (23/31) and 74.19% (23/31), 77.78% (28/36) and 78.38% (29/37); 76.67% (23/30) and 79.31% (23/29), 77.27% (51/66) and 78.79% (52/66), respectively.

**TABLE 2:** Pre-operation subjective diagnosis of abdominal MRI according to pathological results.

Radiologists	Subjective MRI diagnosis	Pathological results		Total
		SPTs	PNETs	
Radiologist 1	SPTs	28	8	36
	PNETs	7	23	30
	Total	35	31	66
Radiologist 2	SPTs	29	8	37
	PNETs	6	23	29
	Total	35	31	66

*SPTs, solid pseudopapillary tumors; PNETs, pancreatic neuroendocrine tumors.*

### Diagnostic Performance of Individual Feature

Each of the 195 features was used to create a ROC curve with respect to the pathological ground truth of PNET or SPT. The feature that had the largest AUC in the primary group was 3 dimension (D)_Gray-level size zone matrix (GLSZM)_Small Zone High Gray-Level Emphasis (SZHGE). Its AUC was 0.762 (95% CI, 0.616–0.909) in the primary group and 0.645 (95% CI, 0.401–0.888) in the test group. The AUCs of other individual features in primary and validation groups were smaller than that of radiomics model (*P* = 0.07 and *P* = 0.11).

### Diagnostic Performance of Radiomics Model

**FIGURE 1:**
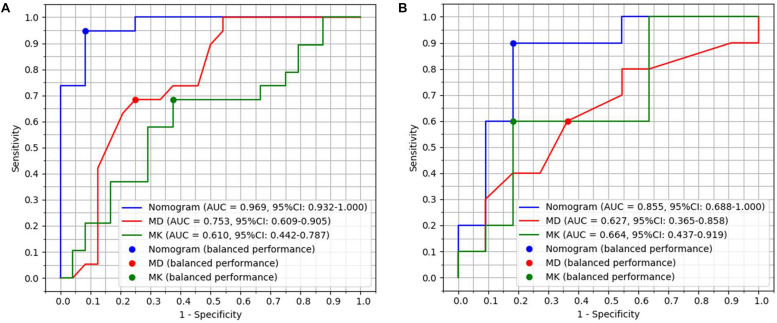
Diagnostic performance with area under curves (AUCs) of radiomics model, MD and MK in primary group with 44 patients and validation group with 22 patients. **(A)** AUCs of radiomics model, MD and MK were 0.97, 0.75, and 0.61 in the primary group, respectively. **(B)** AUCs of radiomics model, MD and MK were 0.86, 0.63, and 0.66 in the validation group, respectively.

**FIGURE 2:**
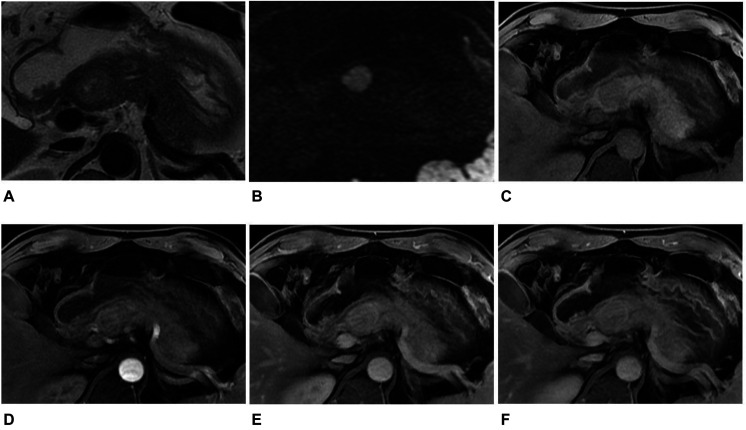
MR images in a 45-year-old man with a non-hypervascular PNET. **(A)** Axial T2-weighted image showed a well-circumscribed high signal intensity tumor in the head of the pancreas. **(B)** The tumor appeared as high signal in DWI with *b* = 1,500 s/mm^2^. **(C–F)** Axial T1-weighted images obtained during plain **(C)**, arterial **(D)**, portal venous **(E)**, and delayed **(F)** phases. The tumors showed enhancement in the arterial, portal venous, and delayed phases when compared with the adjacent parenchyma. The diagnosis of pre-operation MRI was SPT. However, the radiomics analysis showed that the tumor was PNET.

**FIGURE 3:**
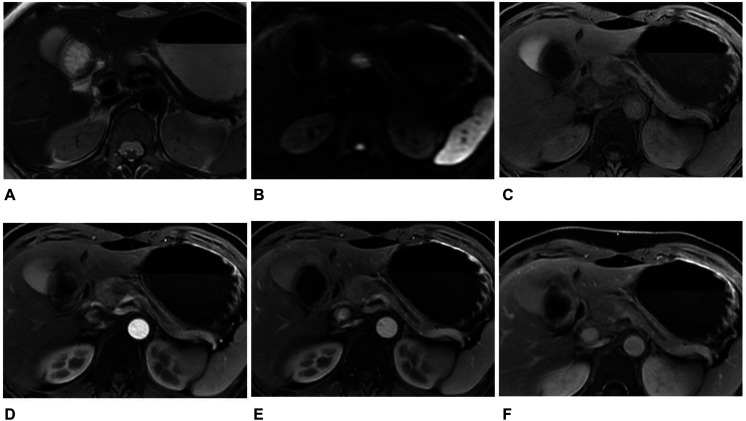
MR images in a 46-year-old woman with an atypical SPT. **(A)** Axial T2-weighted image showed an ill-defined high signal intensity tumor in the neck of the pancreas, accompanied by distal parenchymal ductal dilatation. **(B)** DWI with *b* = 1,500 s/mm^2^ showed a hyperintense tumor. **(C–F)** Axial T1-weighted images obtained during plain **(C)**, arterial **(D)**, portal venous **(E)**, and delayed **(F)** phases. The tumors showed hypovascular enhancement in the arterial phase, gradual enhancement in the portal venous, and progressive hyperenhancement in delayed phases when compared with the adjacent parenchyma. The diagnosis of pre-operation MRI was PNET. However, the radiomics analysis showed that the tumor was SPT.

Seven features of pancreatic tumors were used to construct the radiomics model (Table 3). The radiomics model, which contained radiomics features, age and sex of patients, yielded AUCs of 0.97 [95% CI, 0.932–1.000] and 0.86 [95% CI, 0.688–1.000] in the primary and validation cohort, respectively. The radiomics model achieved a PPV of 90.5% (95% CI, 69.6–98.8%), NPV of 95.7% (95% CI, 78.1–99.9%), sensitivity of 95.0% (95% CI, 75.1–99.9%) and specificity of 91.67% (95% CI, 73.0–99.0%) in primary cohort. The radiomics model resulted in a PPV of 83.3% (95% CI, 51.6–99.9%), NPV of 90.0% (95% CI, 51.0–97.9%), sensitivity of 90.91% (95% CI, 58.7–99.8%) and specificity of 81.82% (95% CI, 48.2–97.7%) in validation cohort. Detailed performance of radiomics model was shown in Figures 1–3 and Table 4.

**TABLE 3:** Parameters of radiomics analysis.

Feature name	Image	Weight	AUC
3D_GLRLM_Long Run Emphasis (LRE)	D_*app*_	−1.0804	0.673 (95%: 0.542–0.804)
3D_GLRLM_Gray-Level Variance (GLV)	K_*app*_	1.7817	0.663 (95%: 0.531–0.795)
3D_NGTDM_Coarseness	K_*app*_	0.5647	0.684 (95%: 0.555–0.813)
3D_NGTDM_Complexity	K_*app*_	−1.6732	0.717 (95%: 0.592–0.842)
Max	T2WI	−1.9308	0.703 (95%: 0.576–0.831)
Kurtosis	T2WI	1.3846	0.628 (95%: 0.491–0.764)
3D_Histogram_Skewness	T2WI	4.7625	0.740 (95%: 0.619–0.860)

*Max, maximum; T2WI, T2 weighted imaging; AUC, area under curve; D, dimension; GLRLM, Gray Level Run Length Matrix; NGTDM, Neighborhood Gray Tone Difference Matrix.*

**TABLE 4:** The AUC, sensitivity, specificity, PPV, and NPV of the radiomics model for discriminating PNETs from SPTs.

	AUC	Sensitivity	Specificity	PPV	NPV
Primary cohort	0.97 (0.790–0.978)	95.00 (75.1–99.9)	91.67 (73.0–99.0)	90.5 (69.6–98.8)	95.7 (78.1–99.9)
Validation cohort	0.86 (0.688–1.000)	90.91 (58.7–99.8)	81.82 (48.2–97.7)	83.30 (51.6–99.9)	90.00 (51.5–97.7)

*AUC, area under curve; PPV, positive predictive value; NPV, negative predictive value.*

The radiomics model had better diagnostic performance than that of MD (*Z* = 3.049, *P* = 0.023) and MK (*Z* = 3.561, *P* = 0.0004) in 66 patients. The accuracy of primary cohort and validation cohort of radiomics model for diagnosing PNETs and SPTs was 95.45% (42/44) and 86.36% (19/22). The accuracy of radiomics model for diagnosing SPTs and PNETs was higher than that of subjective MRI diagnosis (radiologist 1 vs radiomics model, *P* = 0.015; radiologist 2 vs radiomics model, *P* = 0.026).

### Clinical Usefulness

**FIGURE 4:**
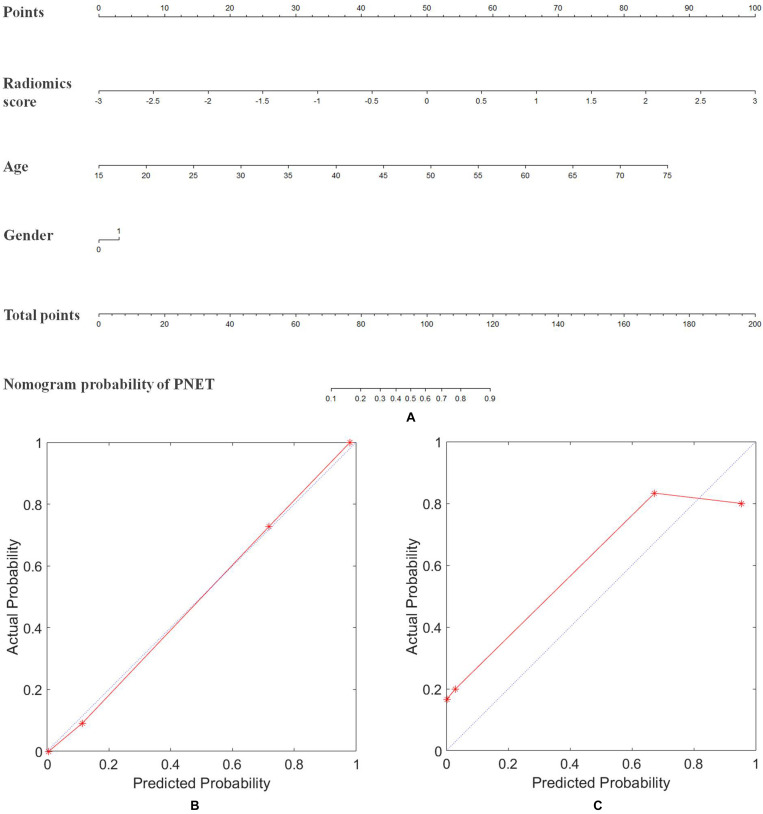
Nomogram of radiomics model for diagnosing the PNET and SPT. **(A)** The developed radiomics nomogram. **(B)** Calibration curves of the radiomics model in the primary cohort. **(C)** Calibration curves of the radiomics model in the validation cohort. Calibration curves depicted the calibration of radiomics model in terms of the agreement between the predicted probability of PNET and the actual outcomes of the PNET. The *y*-axis represented the actual probability of PNET. The *x*-axis represents the predicted probability of PNET. The blue line represents a perfect prediction by an ideal model. The red line shows the performance of the radiomics model based on MRI, age, and gender of patients. The red line was closer to the blue line, which suggested a better prediction.

To provide clinicians with an easy tool, the nomogram based on raidomics analysis, age and gender of patients was developed (Figure 4A). The calibration curves of radiomics model showed excellent performance of this model for clinical use (Figures 4B,C). Patients with pancreatic tumors could benefit from this prediction model.

## Discussion

SPT have good prognosis, with survival rates of 98% after tumor resection (3, 4, 14). PNET patients with complete resection have a survival rate of 90–100%, while patients with incomplete resection have a survival rate ranging from 35 to 75%. In addition, PNET patients with diffuse liver metastases have a 5-years survival rate of 15–25% (15). Pancreas-preserving surgery is the primary treatment strategy, while a formal lymphadenectomy is not carried out for SPTs (2, 14). For PNET, a more aggressive surgical approach of pancreatectomy and extensive lymph node dissections is commonly used to achieve a margin-negative resection. In patients with advanced locoregional or metastatic disease, systemic therapies (such as chemotherapy or targeted agents) could be considered (16). Thus, differentiating PNETs from SPTs using CT and MRI would be useful for surgery planning and the selection of combined treatments.

PNETs are often hyper-vascular and tend to enhance more rapidly and intensely than the normal pancreas, whereas SPTs show progressive non-uniform enhancement that is generally less than that of the normal pancreas on CT and MRI (8, 17). However, previous studies showed that 24% of PNETs manifested hypoenhancement (18, 19). Jeon et al. found non-hypervascularity in the arterial phase in 49% PNET cases (20). In addition, approximately 10–20% of PNETs demonstrated cystic change, which cannot always be readily differentiated from SPTs (16). Although previous studies showed the usefulness of somatostatin receptor imaging with gallium68 (^68^Ga) combined positron emission tomography (PET) in detection and differential diagnosis of PNETs, controversy remains as to its diagnostic performance in high-grade tumors (21, 22). So, applying new abdominal imaging techniques may contribute to reducing the number of PNETs and SPTs false diagnoses.

DKI is an extension of DWI that can be used to evaluate the microstructure features of tissues in a non-Gaussian model. Two quantitative parameters, including D_*app*_ (defining as a good ADC for non-Gaussian bias) and K_*app*_ (representing a deviation from a Gaussian distribution) values, could be extracted by DKI (23). Previous studies reported that DKI should be added to the routine imaging protocol for screening cancer, with good or excellent diagnostic performance in separating malignant cancer from benign lesions (24). With the highest diagnostic accuracy of the diffusion coefficient, the parameters obtained from DKI could predict the microvascular invasion of hepatocellular carcinoma before operation (25). MD derived by DKI has been shown to have a higher diagnostic performance to assess response to electrochemotherapy than conventional DWI parameters and could be used to identify responders and non-responders among patients with pancreatic cancer (26). To the best of our knowledge, so far, only a few reports have utilized DKI to differentiate PNETs and SPTs (10, 26). Jang et al. found that the mean ADC value in SPTs was significantly lower than that in PNETs (6). In our study, the MD of the SPTs was lower than that of PNETs, but there was no significant difference. Contrary to the previous related results on DKI, there was no significant difference in MD and MK between PNETs and SPTs in our study. There were several reasons for this finding. Firstly, MD and MK reflected the heterogeneity of tumors; high-vascular characteristics, calcification, and cystic degeneration commonly presenting in both solid tumors may have similar heterogeneity. Secondly, a small sample size due to the rarity of the tumor may generate the results bias. Thirdly, in this study, the tumor was contoured slice by slice to obtain the entire neoplastic DKI parameters. However, DKI parameters were obtained from the largest tumor section in some related reports (10, 12). We assumed that the DKI quantitative parameters of the entire tumor could provide a more comprehensive tumor characterization.

Previous reports have found that radiomics analysis could sensitively distinguish pancreatic tumors, which confirmed its clinical value (8, 27). We developed and validated a diagnostic, radiomics signature model for differentiation of PNETs and SPTs. The radiomics model incorporating the T2WI and DKI radiomics signature, age, and gender of patients facilitated the pre-operation individualized diagnosis of PNETs and SPTs. Compared with subjective evaluation by radiologists, radiomics analysis could improve the diagnostic performance for distinguishing PNETs from SPTs. The diagnostic accuracies of the radiomics analysis were also higher than that of parameters obtained from DKI.

A reason for the robustness and improved performance of our radiomics model was the use of T2WI. Three T2WI features were used to construct the radiomics signature suggesting that T2WI was a good option for the differentiation between PNETs and SPTs. A recent report has shown the good ability to identify PNETs and SPTs using the morphological features exposed by T2WI with AUC of 0.701 and 0.875 in primary and validation sets, respectively (8). In clinical practice, T2WI could show the cystic degeneration, intra-tumor hemorrhage, and solid component; thus, it played a crucial role in this differentiation. As a result, radiomics features from T2WI could make these characteristics of pancreatic tumors quantitative and add other valuable information related to pancreatic tumor differentiation.

Another explanation for the robustness of our model was the use of DKI derived from DWI. DKI may provide more useful information. As a functional imaging technique, DKI showed strong potential information associated with this differentiation. Among the seven features, one potential feature was obtained from D_*app*,_ and three were extracted from K_*app*_. To the best of our knowledge, no related research on radiomics based on DKI was reported for differentiating pancreatic tumors. DKI showed functional information, such as diffusion, perfusion, heterogeneity, and so on, which may not be reflected by DKI parameters, but could be reflected by the radiomics analysis. In our present study, the use of radiomics based on DKI showed to have good performance for diagnosing PNETs and SPTs with AUC of 0.97 and of 0.86 in the primary and validation cohort, respectively. Our study also showed that the performance of radiomics model for diagnosing SPTs and PNETs was higher than that of subjective MRI diagnosis. Hence, this radiomics-based model could improve the performance and confidence of radiologists in diagnosing PNETs and SPTs and assist doctors in accurately choosing appropriated management.

The present study has several limitations. First, the retrospective nature of this study may have introduced potential selection and verification biases. In addition, as only surgically confirmed tumors were enrolled in our study, our results may not represent the true spectrum of PNETs and SPTs. Second, there was a small sample size due to the rarity of the tumors. A much larger database from multicenter with a considerably larger sample is required to validate the robustness and reproducibility of our radiomics model. Third, our data were acquired with a maximum *b* value of 1,500 s/mm^2^. In general, very high *b*-values (such as 2,000 s/mm^2^) were recommended for the evaluation of DKI. However, various authors have shown that kurtosis effects could be detectable in abdominal imaging when using maximum *b*-values of 800–1,500 s/mm^2^ at 3T (26, 28, 29).

To sum up, radiomics model based on DKI, T2WI, age, and gender of patients may be more valuable than MD and MK for discriminating PNETs and SPTs. This radiomics model could improve diagnostic accuracy and contribute to determining an appropriate treatment strategy for pancreatic tumors. This model could also improve the diagnostic accuracy of differentiating PNETs and SPTs.

## Data Availability Statement

The raw data supporting the conclusions of this article will be made available by the authors, without undue reservation.

## Ethics Statement

The studies involving human participants were reviewed and approved by Peking University Cancer Hospital & Institute. Written informed consent for participation was not provided by the participants’ legal guardians/next of kin because this was a retrospective study approved by our institutional review board, and informed consent was waived.

## Author Contributions

Y-JS and Y-SS: study conception and design. Y-LL, Y-YW, and X-BQ: data collection and analysis. Y-JS and H-TZ: manuscript writing. X-TL and H-TZ: statistical analysis and radiomics model construction. X-YZ, X-TL, and Y-SS: manuscript revision. All authors reviewed the manuscript review and gave final approval of manuscript. Y-JS, H-TZ, and Y-LL contributed equally to this article.

## Conflict of Interest

The authors declare that the research was conducted in the absence of any commercial or financial relationships that could be construed as a potential conflict of interest.

## Footnotes

1 https://itk.org/
